# Intra-Omicron Reinfection with JN.1.16 and NB.1.8.1 in a Preterm Infant: First NB.1.8.1 Detection in Tunisia—A Case Report

**DOI:** 10.3390/microorganisms14051009

**Published:** 2026-04-30

**Authors:** Zaineb Hamzaoui, Sana Ferjani, Ameni Sallemi, Salma Abid, Amal Miraoui, Ichrak Landolsi, Latifa Charaa, Khaled Menif, Lamia Kanzari, Ilhem Boutiba-Ben Boubaker

**Affiliations:** 1 Research Laboratory “Antimicrobial Resistance” LR99ES09, Faculty of Medicine of Tunis, University of Tunis El Manar, Tunis 1007, Tunisia; sana.ferjani@rns.tn (S.F.); asalma680@rns.tn (S.A.); lamia.kanzari@fmt.utm.tn (L.K.); ilhem.boutiba@rns.tn (I.B.-B.B.); 2 Charles Nicolle Hospital, Laboratory of Microbiology, National Influenza Centre, National Reference Laboratory for Influenza and Other Respiratory Viruses, Tunis 1006, Tunisia; ichra99bio@gmail.com (I.L.); latifa.charaa@gmail.com (L.C.); 3 Laboratory of Clinical Pharmacology, National Centre Chalbibelkahia of Pharmacovigilance, Tunis 1007, Tunisia; amenisallemipharmaco@gmail.com; 4 Pediatric Intensive Care Unit, Béchir Hamza Children’s Hospital, Tunis 1006, Tunisia; amal.miraoui@fmt.utm.tn (A.M.); khaled.menif@fmt.utm.tn (K.M.)

**Keywords:** SARS-CoV-2, Omicron, NB.1.8.1, JN.1.16, preterm infant, reinfection, whole-genome sequencing, ACE2-binding affinity

## Abstract

Highly mutated Omicron sub-lineages JN.1 and NB.1.8.1 harbor extensive spike changes, but their impact in preterm infants is poorly documented. We report a preterm male infant with three hospitalizations in seven weeks: severe SARS-CoV-2 ARDS at 40 days of life (DOL 40) requiring ventilation caused by JN.1.16, HCoV-OC43 infection at DOL 65, and a mild SARS-CoV-2 reinfection at DOL 87 due to NB.1.8.1, the first detection of this variant in Tunisia. Spike analysis showed a shared JN.1 backbone but distinct N-terminal and receptor-binding domain changes, supporting intra-Omicron reinfection driven by antigenic divergence and immature immunity and underscoring the value of pediatric genomic surveillance, including phylogenetic placement of case genomes within local Omicron diversity.

## 1. Introduction

Since late 2021, the Omicron variant of SARS-CoV-2 has remained globally dominant while continuously diversifying through antigenic drift and occasional recombination, generating successive waves of sub-lineages with enhanced transmissibility and immune escape. Recent Omicron evolution has been marked by the emergence of the highly divergent BA.2.86 branch and its descendants, reflecting substantial spike remodeling, particularly across the N-terminal domain (NTD) and receptor-binding domain (RBD), enabling recurrent infections despite widespread population immunity [1].

Within this BA.2.86 lineage, JN.1 (BA.2.86.1.1) showed a clear growth advantage and was classified by the World Health Organization (WHO) as a Variant of Interest (VOI), subsequently seeding numerous expanding descendant lineages globally [1,2]. In 2025, further JN.1-derived lineages and recombinants continued to emerge. NB.1.8.1, designated by WHO (and assessed by ECDC) as a Variant Under Monitoring (VUM), has been associated with increasing detections and carries additional spike substitutions that may enhance fitness and immune evasion [3,4,5].

Infants in the first months of life, who are typically not vaccine-eligible, represent a vulnerable group, with hospitalization rates during Omicron periods remaining substantial in surveillance reports. Clinical series indicate that many infections in young infants are mild, yet severe disease requiring intensive care can occur, particularly among premature infants and those with comorbidities [6].

Because publicly available genomic data from Tunisia remain limited, placing these episode viruses in their national circulation context is essential to interpret the reinfection and to document the timing of introduction of newly emerging JN.1-descendant lineages.

Here, we report a preterm infant who experienced severe SARS-CoV-2 infection with JN.1.16, an intermediate HCoV-OC43 episode, and a short-interval SARS-CoV-2 reinfection with NB.1.8.1 within seven weeks. We provide whole-genome sequencing for both SARS-CoV-2 episodes, phylogenetic placement within a Tunisian Omicron dataset, and comparative spike mutation profiling.

## 2. Materials and Methods

### 2.1. Clinical Data

A male infant born at 35 weeks with left ureteropyelocaliceal dilatation was hospitalized in the Bechir Hamza Children’s Hospital pediatric intensive care unit (PICU). Clinical and epidemiologic data were collected from medical records and surveillance forms.

### 2.2. Virus Detection

Nasopharyngeal swabs were analyzed at the National Influenza and Respiratory Viruses Laboratory, Charles Nicolle Hospital. A single nasopharyngeal swab was collected and tested for each respiratory episode. No other respiratory specimen types were analyzed. RNA extraction was performed using the Chemagic™ automate and the Viral RNA 300 Kit H96 (PerkinElmer, Hamburg, Germany), according to the manufacturer’s instructions. SARS-CoV-2 and influenza A/B were tested by the CDC Flu SC2 multiplex assay, and all samples by the FTD™ Respiratory Pathogens 21 panel (Fast Track Diagnostics; Siemens Healthineers, Esch-sur-Alzette, Luxembourg) [7]. Appropriate controls were included.

### 2.3. Whole-Genome Sequencing

SARS-CoV-2–positive samples were sequenced using Illumina RNA Prep with Enrichment and a respiratory virus panel on an iSeq 100 platform. Reads were filtered, trimmed, mapped to Wuhan-Hu-1 and used for consensus generation. Lineages were assigned with Nextclade and confirmed with Pangolin; spike/non-spike mutations were extracted from Nextclade reports. Consensus genomes were uploaded to GISAID AudacityInstant for phylogenetic context and geographic visualization. For NB.1.8.1, closest neighbors were further analyzed in Nextstrain for time-resolved phylogeny and phylogeographic mapping.

### 2.4. Mutational Analysis

Spike changes were grouped by region (NTD, RBD, S1/S2, S2) and annotated for predicted functional effects (immune escape, ACE2 binding, fusogenicity, replication fitness). RBD ACE2-binding affinity scores from Nextclade were used as qualitative indicators and visualized on the BA.2.86/JN.1 phylogeny.

### 2.5. Genomic Context

To contextualize the case within contemporaneous SARS-CoV-2 circulation in Tunisia, we retrieved all Tunisian genomes publicly available in GISAID (EpiCoV) with collection dates between 13 June and 25 August 2025 (data exported on 27 December 2025). For each genome, metadata (collection date and Pango lineage as reported in GISAID at the time of export) were extracted and summarized.

### 2.6. Phylogenetic Analysis

To place the two SARS-CoV-2 genomes generated in this case report (episodes 1 and 3) in an evolutionary context, we compiled a contextual dataset of Tunisian Omicron genomes retrieved from GISAID (collection dates from 1 November 2021 to 30 November 2025). In total, 106 sequences were analyzed, including the two study genomes, 103 Tunisian genomes from GISAID, and one BA.2.86 reference genome (Nextclade dataset). Sequences were aligned with Nextclade against Wuhan-Hu-1 (NC_045512.2), and the resulting alignment was exported for phylogenetic inference. A maximum-likelihood tree was inferred using IQ-TREE (GTR + G model), with node support assessed by 1000 ultrafast bootstrap replicates (UFboot) and 1000 SH-aLRT replicates. The tree was visualized and annotated in iTOL, highlighting the two genomes from this study; densely sampled clades were collapsed for readability. The list of all sequences included (GISAID accession IDs and metadata) is provided in Table S2.

## 3. Results

### 3.1. Clinical and Virological Course of Three Respiratory Episodes

Three diagnostic nasopharyngeal swabs (one per episode; *n* = 3) were collected and tested. During the first episode, at 40 days of life (DOL 40), the preterm infant presented fever, cough and signs of acute respiratory distress. He was admitted to the PICU with acute respiratory distress syndrome and required invasive mechanical ventilation with prone positioning. Real-time RT-PCR detected SARS-CoV-2 with a low cycle threshold (Ct ≈ 20) consistent with high viral load. No bacterial bloodstream infection was documented. The FTD™ Respiratory Pathogens 21 panel was negative for HCoV-OC43 and all other tested respiratory viruses during episode 1. The clinical course was favorable under intensive care support, and the infant was extubated and discharged (Table 1). The mother had not received COVID-19 vaccination during pregnancy, and the infant had not received any COVID-19 vaccine.

The second episode (DOL 65) occurred three weeks after discharge. The infant was rehospitalized for recurrent fever (38.5 °C) and cough. A nasopharyngeal swab was collected on the day of admission; however, due to weekend laboratory workflow, the sample was processed and tested three days later. SARS-CoV-2 RT-PCR was negative, whereas multiplex RT-PCR identified HCoV-OC43 with a Ct value of 23. Respiratory distress was moderate, no mechanical ventilation was required, and the infant improved with supportive care (Table 1).

The third episode (DOL 87) took place 25 days after the second hospitalization. The infant again presented with cough and low-grade fever but without hypoxemia. Multiplex RT-PCR was positive for SARS-CoV-2 with a Ct value of 19. The FTD™ Respiratory Pathogens 21 panel was negative for HCoV-OC43 and all other tested respiratory viruses during episode 3. The clinical course remained mild, with no need for mechanical ventilation; the infant was managed in the infant medicine ward and discharged after clinical improvement (Table 1).

### 3.2. Genomic Features of the Two SARS-CoV-2 Episodes

High-quality genomes were submitted to GISAID (EPI_ISL_20142345, EPI_ISL_20207111) and linked to BioProject PRJNA1359743. Nextclade/Pangolin classified the first genome as JN.1.16 (Omicron 23I) and the reinfection genome as NB.1.8.1 (XDV.1.5.1.1.8.1, clade 25B).

AudacityInstant showed JN.1.16 clustering with European/North American JN.1.16/LF.7 sequences, closest to a Trieste, Italy isolate (EPI_ISL_20126820, distance = 3, score 0.988).

**Figure 1:**
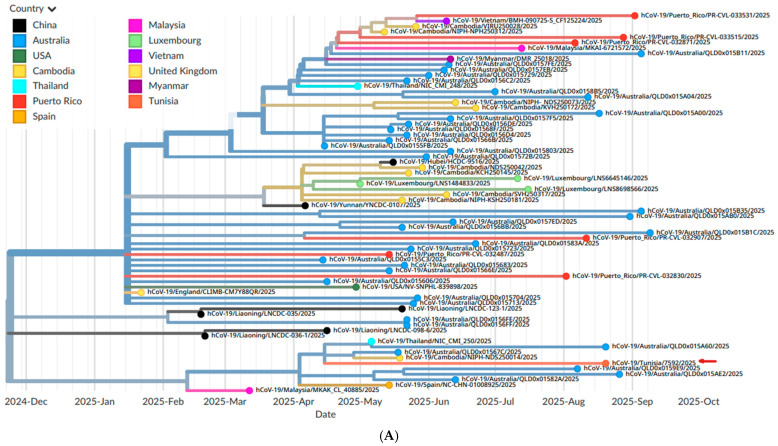

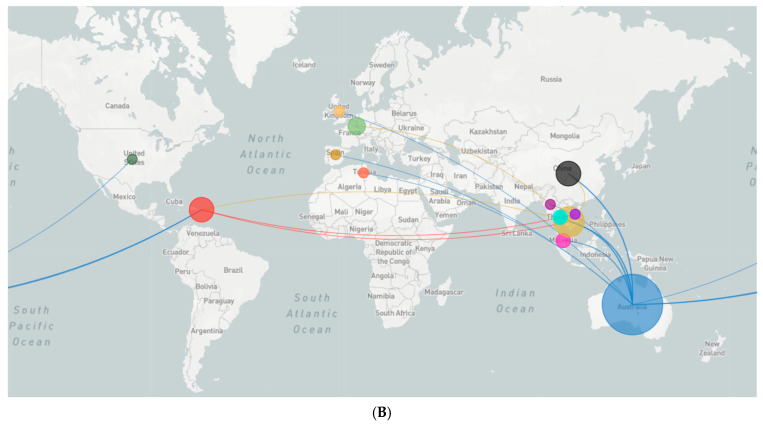
International phylogenetic and geographic context of the Tunisian NB.1.8.1 genome. (**A**). Time-resolved Nextstrain phylogeny of the Tunisian NB.1.8.1 genome (The Tunisian sequence hCoV-19/Tunisia/7592/2025 is marked with a red arrow) and its closest neighbors. Branches and tip circles are colored by country of sampling. The Tunisian sequence clusters within an NB.1.8.1/XDV clade largely composed of Australian genomes, with additional related sequences from Asia, Europe, North America and the Caribbean. (**B**). Phylogeographic map of the same set of NB.1.8.1/XDV genomes. Circle area is proportional to the number of sequences per country, and colors indicate the country of sampling. The large circle over Australia denotes that most closely related genomes were detected there, while smaller circles over Puerto Rico, the United States, Tunisia and several European and Asian countries illustrate broader global spread. The Tunisian NB.1.8.1 genome is shown as a single node in North Africa connected to these international clusters.

NB.1.8.1 clustered within an XDV clade dominated by Australian genomes, with additional related sequences from Asia, Europe, North America and the Caribbean (Figure 1A,B).

### 3.3. Comparative Mutational Signatures of JN.1.16 and NB.1.8.1

**Figure 2:**
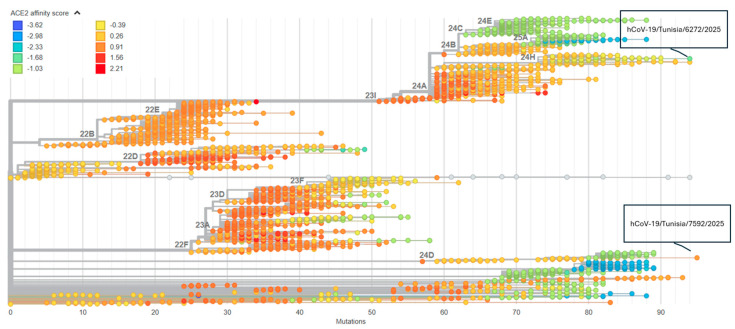
Global BA.2.86/JN.1 phylogeny colored by predicted ACE2-binding affinity. Each circle represents a BA.2.86/JN.1-descendant genome from the Nextclade dataset, positioned by the total number of amino-acid substitutions relative to Wuhan-Hu-1 (*x*-axis) and colored according to the predicted RBD ACE2-binding score (from blue, lower affinity, to red, higher affinity). The infant JN.1.16 isolate hCoV−19/Tunisia/6272/2025 (green) and NB.1.8.1/XDV isolate hCoV−19/Tunisia/7592/2025 (orange) are labeled, showing ACE2-affinity scores within the range of contemporaneous JN.1-descendant lineages.

Both genomes shared the characteristic JN.1 spike backbone (NTD deletions, F486P, N679K/P681R, Q954H, N969K). JN.1.16 displayed additional NTD/RBD escape mutations (N440K, L452W, E484K, F456L, N460K, Q498R/N501Y/Y505H), while NB.1.8.1 lacked F456L and N460K but gained A264D, G339H, K356T, A688T, K1149R (Supplementary Table S1). In the Nextclade BA.2.86/JN.1 phylogeny, NB.1.8.1 showed a slightly higher predicted ACE2 affinity than JN.1.16 (Figure 2). Non-spike changes (NSP12 P323L, N P13L + R203K/G204R) were shared.

### 3.4. Temporal Context of Lineage Circulation in Tunisia During the Case Episodes

**Figure 3:**
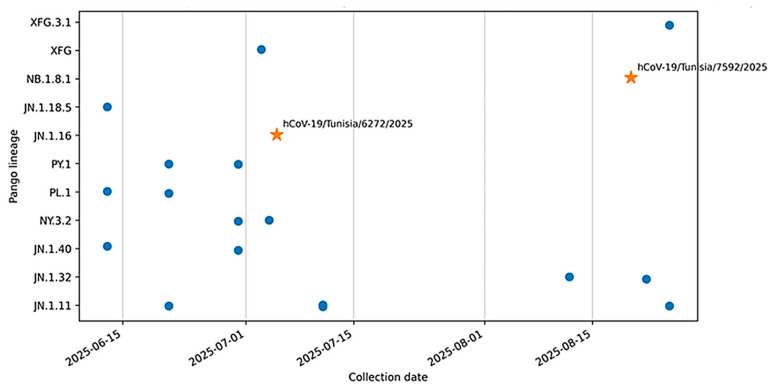
Temporal distribution of SARS-CoV-2 Pango lineages in Tunisia based on GISAID genomes (June–August 2025). Each point represents one genome positioned by collection date (*x*-axis) and Pango lineage (*y*-axis). Stars indicate the two case genomes (IDs hCoV-19/Tunisia/6272/2025 and hCoV-19/Tunisia/7592/2025), corresponding to JN.1.16 (episode 1) and NB.1.8.1 (episode 3). Lineage assignments are shown as reported in GISAID at the time of data export.

Among Tunisian genomes publicly available in GISAID during 13 June–25 August 2025 (*n* = 19), JN.1-descendant lineages predominated. The most frequently reported lineage was JN.1.11 (4/19), followed by JN.1.32, JN.1.40, PL.1, PY.1, and NY.3.2 (each 2/19), whereas JN.1.16 was observed in early July (1/19). Notably, no additional NB.1.8.1-related genomes were observed among other contemporaneous Tunisian sequences beyond the reinfection strain (Figure 3).

### 3.5. Phylogenetic Placement of the Two SARS-CoV-2 Episodes

**Figure 4:**
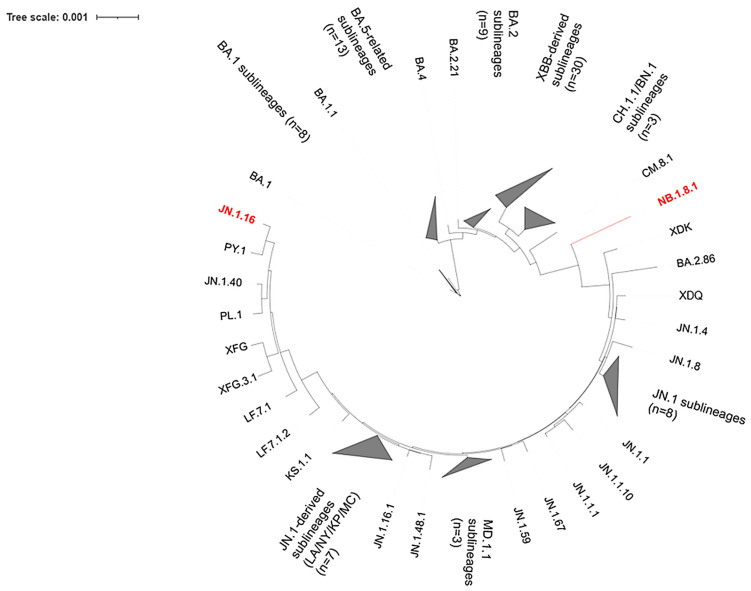
Maximum-likelihood phylogeny of Omicron lineages placing the episode 1 and episode 3 genomes in a Tunisian genomic context. The two SARS-CoV-2 genomes generated in this study (episodes 1 and 3) are highlighted in red. Gray triangles indicate collapsed, densely sampled clades for readability; labels denote the lineage group and the number of sequences (n) included in each collapsed clade. Branch lengths are drawn to scale (substitutions per site), and the scale bar is shown on the figure. The full list of genomes and associated metadata used to build the tree is provided in Table S2.

In the maximum-likelihood phylogeny (Figure 4), the episode 1 genome fell within the JN.1-derived diversity and clustered in the JN.1.16 clade, whereas the episode 3 genome clustered within the NB.1.8.1 lineage. Thus, both genomes are positioned within the BA.2.86/JN.1-related diversification of Omicron, but they occupy distinct sub-lineage placements consistent with two separate episodes. For clarity of presentation, densely sampled Omicron clades were collapsed into triangles in Figure 4 to improve readability without altering the underlying tree topology.

## 4. Discussion

In this vulnerable preterm infant, three closely spaced coronavirus-associated respiratory episodes illustrate how immature host defenses and evolving SARS-CoV-2/seasonal coronavirus epidemiology can interact to produce a complex clinical course. The first episode, at 40 days of life, was dominated by severe SARS-CoV-2 pneumonia with acute respiratory distress syndrome requiring invasive mechanical ventilation, in line with reports that prematurity and underlying neonatal comorbidities substantially increase the risk of severe COVID-19 and intensive care admission compared with term infants [3,4]. Large reviews of neonatal SARS-CoV-2 infection show that most neonates have asymptomatic or mild disease, but a non-negligible subset—often preterm—develops respiratory failure and requires ventilatory support [5], as in our case.

Neither the mother nor the infant had received COVID-19 vaccination, and the mother was not vaccinated during pregnancy; therefore, vaccine failure cannot be assessed in this case. This point is nonetheless relevant because infants in the first months of life are not vaccine-eligible in most settings and largely rely on maternally transferred antibodies for early protection [8]. Large observational studies have shown that maternal mRNA COVID-19 vaccination during pregnancy is associated with a reduced risk of COVID-19–related hospitalization in infants aged <6 months, although effectiveness appears attenuated during Omicron-predominant periods, consistent with immune escape [9]. In our patient, the absence of maternal vaccination (and thus lack of vaccine-induced transplacental antibodies) may have contributed to susceptibility in early life, and this may be further amplified by prematurity, which can limit the magnitude of placental IgG transfer. Importantly, we interpret this as a plausibility consideration rather than evidence of causality. Finally, the occurrence of genetically confirmed reinfection within a short interval is also biologically consistent with the well-described ability of Omicron subvariants to cause rapid reinfections, particularly in individuals without prior vaccine- or infection-derived immunity [10,11].

The high viral load (Ct ≈ 20) during this first episode is consistent with pediatric data linking lower Ct values to symptomatic infection and greater need for respiratory support in infants [12]. The absence of documented bacterial bloodstream infection or viral co-infection agrees with recent series showing very low rates of invasive bacterial infection in febrile young infants with SARS-CoV-2, supporting the notion that severe respiratory disease can be driven primarily by the virus itself in this age group [13].

The second hospitalization, three weeks after discharge, was virologically distinct, with SARS-CoV-2 negativity and identification of HCoV-OC43. Seasonal HCoV-OC43 is among the most frequent coronaviruses in early childhood and is increasingly recognized as a cause of bronchiolitis and lower respiratory tract infection, with prematurity and chronic conditions associated with more severe courses [14]. In our patient, the second episode manifested as moderate respiratory distress without the need for mechanical ventilation, compatible with the generally mild-to-moderate severity described in immunocompetent children [15]. The clear switch from SARS-CoV-2 to OC43 between episodes, with intervening SARS-CoV-2 negativity, supports sequential, etiologically distinct infections rather than simple prolonged shedding.

The third episode represents a true SARS-CoV-2 reinfection in a very short interval—approximately seven weeks after the first infection—again with a low Ct value around 19 but a much milder clinical picture. WGS confirmed that the two SARS-CoV-2 episodes were caused by distinct Omicron sub-lineages, JN.1.16 and NB.1.8.1, fulfilling genomic criteria for reinfection rather than persistent RNA detection. This pattern is coherent with accumulating evidence that Omicron sub-lineages have marked immune escape properties and can reinfect individuals within weeks to months despite recent infection or vaccination, including in pediatric populations [16]. Reports of early Omicron reinfections, sometimes within 60 days, particularly concern unvaccinated young individuals exposed to antigenically divergent sub-lineages. Our observation adds to this literature by documenting short-interval reinfection in a preterm infant with careful clinical and genomic characterization [17].

Interestingly, disease severity decreased across the three episodes, from life-threatening ARDS during the primary SARS-CoV-2 infection to moderate OC43 disease and finally a mild SARS-CoV-2 reinfection despite similarly high viral loads. Several mechanisms may underline this pattern. Even in very young children, natural infection can rapidly prime innate and adaptive immune responses, and prior infection has been associated with reduced risk of severe disease upon reinfection, even in the Omicron era, although protection against infection is only partial and wanes over time. Repeated viral exposures in early life may also modulate mucosal and interferon responses, leading to more efficient control at subsequent encounters. The interposition of an OC43 infection could have induced heterologous immune activation, but the direction and magnitude of cross-protection between endemic HCoVs and SARS-CoV-2 remain unclear [18].

Genomic sequencing confirmed that the first episode was caused by JN.1.16 within the JN.1 umbrella, a BA.2.86-descendant lineage that became globally dominant because of marked transmissibility and immune escape, whereas the reinfection episode was due to NB.1.8.1 (XDV.1.5.1.1.8.1), a recombinant Omicron lineage with a documented growth advantage. AudacityInstant and Nextstrain analyses showed that the JN.1.16 genome clustered with JN.1.16/LF.7 sequences from Europe and North America, while the NB.1.8.1 genome fell within an NB.1.8.1/XDV clade dominated by Australian sequences with additional genomes from Asia, Europe, North America and the Caribbean. This pattern supports acquisition of both viruses from already globally circulating JN.1-family lineages rather than local emergence and indicates that the Tunisian NB.1.8.1 genome represents the first documented detection of this variant in Tunisia and, to our knowledge, in North Africa.

To place this intra-Omicron reinfection in a Tunisia-specific context, we reviewed all Tunisian SARS-CoV-2 genomes publicly available in GISAID collected between 13 June and 25 August 2025 (*n* = 19; export date: 27 December 2025). Available sequences were dominated by JN.1-descendant lineages, and the episode 1 genome (JN.1.16; DOL 40) was temporally consistent with contemporaneous JN.1-derived circulation in the country. In contrast, the reinfection strain detected at DOL 87 belonged to the NB.1.8.1 lineage and was not identified among other contemporaneous Tunisian sequences beyond our case, supporting a likely recent introduction in Tunisia, while acknowledging limited national sequencing density. Notably, within this limited dataset, JN.1.16 was recorded in early July, whereas NB.1.8.1 was only recorded in late August, consistent with a temporal shift in detected lineages between the two SARS-CoV-2 episodes.

Using a broader Tunisian Omicron dataset (1 November 2021–30 November 2025; *n* = 106, including the two study genomes), maximum-likelihood phylogenetic analysis placed the episode 1 genome within the JN.1.16 clade and the episode 3 genome within NB.1.8.1. This supports two genetically distinct Omicron infections rather than persistent viral shedding.

Nosocomial acquisition was considered given the infant’s repeated hospitalizations and PICU stay. However, both SARS-CoV-2 episodes were detected on diagnostic samples collected at/near admission (episode 1 sampled the day after admission; episode 3 sampled on the day of admission), and the infant had been discharged home between episodes, arguing against infection acquired during the preceding hospitalization. Nevertheless, healthcare-associated transmission cannot be completely excluded in the absence of contemporaneous sequencing from other patients/healthcare workers and detailed exposure tracing.

The comparative mutational profiles of JN.1.16 and NB.1.8.1 in this infant illustrate how Omicron evolution balances immune escape and preservation of efficient ACE2 usage. Both genomes share the heavily mutated BA.2.86/JN.1 spike backbone, including NTD deletions, the hallmark F486P substitution and the N679K/P681R motif at the S1/S2 junction, which together have been associated with strong immune evasion and maintained ACE2 affinity [19]. On this shared scaffold, JN.1.16 carries a dense constellation of additional NTD and RBD changes consistent with an escape-maximized profile, whereas NB.1.8.1 lacks some of these aggressive escape mutations but acquires alternative NTD and RBD substitutions and shows a higher predicted ACE2-binding score in the Nextclade phylogeny. This fits with experimental data describing NB.1.8.1 and related XDV sub-lineages as fitness-optimized recombinants with modestly increased ACE2 binding and cell entry while retaining substantial immune escape [20]. Direct lineage-specific neutralization data comparing sera primed by JN.1.16 versus NB.1.8.1 are limited. However, available antigenic and neutralization data for late JN.1-descendant and recombinant lineages are consistent with reduced neutralization of emerging variants relative to homologous JN.1-lineage antigens/sera and support the plausibility of partial immune escape. Therefore, the short-interval reinfection observed here is biologically plausible, as antigenic differences across NTD/RBD between JN.1 sub-lineages and recombinant XDV-related lineages may reduce cross-protection, especially in a preterm infant with immature immunity [2,6,21,22].

The two genomes shared many non-spike changes, such as NSP12 P323L and the nucleocapsid signature P13L with R203K/G204R, placing both viruses inside the conventional Omicron framework and indicating that spike is primarily responsible for the major functional variations between episodes.

## 5. Conclusions

This case shows how rapidly evolving Omicron lineages, including recombinants such as NB.1.8.1, can exploit gaps in immunity and cause sequential infections in highly vulnerable infants. It highlights the importance of pediatric genomic surveillance to detect emerging variants and relate them to clinical phenotypes and supports close follow-up of preterm infants with severe early SARS-CoV-2 disease and early reinfection.

## Figures and Tables

**Table 1:** Clinical and virological characteristics of three hospitalizations in a premature infant: day of life at sampling, main symptoms, SARS-CoV-2 Ct values, identified respiratory viruses and need for mechanical ventilation.

Episode	Admission Date	Sample Date	Day of Life at Sampling	Main Symptoms	Virus Detected	Ct Value	ICU Mechanical Ventilation	Sample ID
1	3 July 2025	4 July 2025	40	Fever 38.5 °C, cough; ARDS	SARS-CoV-2 (Omicron JN.1.16)	20	Yes	hCoV-19/Tunisia/6272/2025
2	26 July 2025	29 July 2025	65	Fever 38.5 °C, cough	HCoV-OC43	23	Not documented	HCoV-OC43/Tunisia/7041/2025
3	20 August 2025	20 August 2025	87	Fever 37.1 °C, cough	SARS-CoV-2 (Omicron NB.1.8.1)	19	Not documented	hCoV-19/Tunisia/7592/2025

ARDS, Acute Respiratory Distress Syndrome. Each episode corresponds to one diagnostic nasopharyngeal swab. For episode 2, the swab was collected on the day of admission; ‘Sample date’ indicates the testing date (delayed due to weekend workflow).

## Data Availability

Raw sequencing reads generated in this study have been deposited in the NCBI Sequence Read Archive (SRA) under the BioProject PRJNA1359743. The corresponding consensus genome sequences are available from the GISAID database under accession numbers EPI_ISL_20142345 and EPI_ISL_20207111, in accordance with GISAID’s data access policies. De-identified clinical and laboratory metadata underlying the reported findings are not publicly available due to privacy and institutional restrictions but can be obtained from the corresponding author upon reasonable request and subject to applicable ethical and regulatory approvals.

## Supplementary Materials

The following supporting information can be downloaded at: https://www.mdpi.com/article/10.3390/microorganisms14051009/s1; Table S1: Key spike mutations and functional annotations in JN.1.16 (Episode 1) and NB.1.8.1 (Episode 3); Table S2: Tunisian SARS-CoV-2 Omicron genomes included in the phylogenetic analysis (GISAID metadata; *n* = 106, 1 November 2021–30 November 2025).

## Abbreviations

The following abbreviations are used in this manuscript:

SARS-CoV-2	Severe Acute Respiratory Syndrome Coronavirus 2
COVID-19	Coronavirus Disease 2019
ARDS	Acute Respiratory Distress Syndrome
HCoV-OC43	Human Coronavirus OC43
WHO	World Health Organization
PICU	Pediatric Intensive Care Unit
RT-PCR	Reverse-Transcription Polymerase Chain Reaction
Ct	Cycle threshold
WGS	Whole-Genome Sequencing
NTD	N-terminal Domain (of spike)
RBD	Receptor-Binding Domain (of spike)
S1/S2	S1/S2 cleavage site of the spike protein
ACE2	Angiotensin-Converting Enzyme 2
CDC	Centers for Disease Control and Prevention
FTD	Fast Track Diagnostics
RNA	Ribonucleic Acid
NSP12	Non-Structural Protein 12
NCBI	National Center for Biotechnology Information
SRA	Sequence Read Archive

## References

[1] Planas D., Staropoli I., Michel V., Lemoine F., Donati F., Prot M., Porrot F., Guivel-Benhassine F., Jeyarajah B., Brisebarre A. (2024). Distinct Evolution of SARS-CoV-2 Omicron XBB and BA.2.86/JN.1 Lineages Combining Increased Fitness and Antibody Evasion. Nat. Commun..

[2] WHO (2025). WHO TAG-VE Risk Evaluation for SARS-CoV-2 Variant Under Monitoring: NB.1.8.1.

[3] Nyholm S., Edner A., Myrelid Å., Janols H., Dörenberg R., Diderholm B. (2020). Invasive Mechanical Ventilation in a Former Preterm Infant with COVID-19. Acta Paediatr..

[4] Sarhan M.A., Casalino M., Paopongsawan P., Gryn D., Kulkarni T., Bitnun A., Gauda E.B. (2022). SARS-CoV-2 Associated Respiratory Failure in a Preterm Infant and the Outcome after Remdesivir Treatment. Pediatr. Infect. Dis. J..

[5] Raschetti R., Vivanti A.J., Vauloup-Fellous C., Loi B., Benachi A., De Luca D. (2020). Synthesis and Systematic Review of Reported Neonatal SARS-CoV-2 Infections. Nat. Commun..

[6] Guo C., Yu Y., Liu J., Jian F., Yang S., Song W., Yu L., Shao F., Cao Y. (2025). Antigenic and Virological Characteristics of SARS-CoV-2 Variants BA.3.2, XFG, and NB.1.8.1. Lancet Infect. Dis..

[7] Siemens Healthcare Diagnostics Inc. FTD Respiratory Assay Solutions (Includes FTD Respiratory Pathogens 21; IFU 11414180_en Rev. B). https://cdn0.scrvt.com/39b415fb07de4d9656c7b516d8e2d907/b6707b45e1e19301/43f395d77a40/ous-ftd-respiratory-assay-solutions-brochure-0720-final.pdf.

[8] Cinicola B., Conti M.G., Terrin G., Sgrulletti M., Elfeky R., Carsetti R., Fernandez Salinas A., Piano Mortari E., Brindisi G., De Curtis M. (2021). The Protective Role of Maternal Immunization in Early Life. Front. Pediatr..

[9] Halasa N.B., Olson S.M., Staat M.A., Newhams M.M., Price A.M., Pannaraj P.S., Boom J.A., Sahni L.C., Chiotos K., Cameron M.A. (2022). Maternal Vaccination and Risk of Hospitalization for Covid-19 among Infants. N. Engl. J. Med..

[10] Rodríguez-Grande C., Estévez A., Palomino-Cabrera R., Molero-Salinas A., Peñas-Utrilla D., Herranz M., Sanz-Pérez A., Alcalá L., Veintimilla C., Catalán P. (2023). Early SARS-CoV-2 Reinfections Involving the Same or Different Genomic Lineages, Spain. Emerg. Infect. Dis..

[11] Vera-Lise I., Dominik E., Elisabeth R., Kerstin H., Raffael F., Angelika X., Tibor A., Jusztina B., Ursula K., Jochen H. (2022). Rapid reinfections with different or same Omicron SARS-CoV-2 sub-variants. J. Infect..

[12] Bankers L., O’Brien S.C., Tapay D.M., Ho E., Armistead I., Burakoff A., Dominguez S.R., Matzinger S.R. (2024). SARS-CoV-2 Disease Severity and Cycle Threshold Values in Children Infected during Pre-Delta, Delta, and Omicron Periods, Colorado, USA, 2021–2022. Emerg. Infect. Dis..

[13] Hernández-Bou S., Trenchs V., Diego P., Seguí A., Luaces C. (2023). Bacterial Coinfection in Young Febrile Infants with SARS-CoV-2 Infection. Eur. J. Pediatr..

[14] Jo K.J., Choi S.-H., Oh C.E., Kim H., Choi B.S., Jo D.S., Park S.E. (2022). Epidemiology and Clinical Characteristics of Human Coronaviruses-Associated Infections in Children: A Multi-Center Study. Front. Pediatr..

[15] Abu Shanap M., Sughayer M., Alsmadi O., Elzayat I., Al-Nuirat A., Tbakhi A., Sultan I. (2022). Factors That Predict Severity of Infection and Seroconversion in Immunocompromised Children and Adolescents with COVID-19 Infection. Front. Immunol..

[16] Shen H., Chen D., Li C., Huang T., Ma W. (2024). A Mini Review of Reinfection with the SARS-CoV-2 Omicron Variant. Health Sci. Rep..

[17] Chemaitelly H., Ayoub H.H., Coyle P., Tang P., Yassine H.M., Al-Khatib H.A., Smatti M.K., Hasan M.R., Al-Kanaani Z., Al-Kuwari E. (2022). Protection of Omicron Sub-Lineage Infection against Reinfection with Another Omicron Sub-Lineage. Nat. Commun..

[18] Guo L., Wang Y., Kang L., Hu Y., Wang L., Zhong J., Chen H., Ren L., Gu X., Wang G. (2021). Cross-Reactive Antibody against Human Coronavirus OC43 Spike Protein Correlates with Disease Severity in COVID-19 Patients: A Retrospective Study. Emerg. Microbes Infect..

[19] Liu Y., Zhao X., Shi J., Wang Y., Liu H., Hu Y.-F., Hu B., Shuai H., Yuen T.T.-T., Chai Y. (2024). Lineage-Specific Pathogenicity, Immune Evasion, and Virological Features of SARS-CoV-2 BA.2.86/JN.1 and EG.5.1/HK.3. Nat. Commun..

[20] Uriu K., Okumura K., Uwamino Y., Chen L., Tolentino J.E., Asakura H., Nagashima M., Sadamasu K., Yoshimura K., Ito J. (2025). Virological Characteristics of the SARS-CoV-2 NB.1.8.1 Variant. Lancet Infect. Dis..

[21] Abbad A., Lerman B., Ehrenhaus J., Monahan B., Singh G., Wilson A., Slamanig S., Aracena A., Lyttle N., Nardulli J. (2025). Antibody Responses to SARS-CoV-2 Variants LP.8.1, LF.7.1, NB.1.8.1, XFG and BA.3.2 Following KP.2 Monovalent mRNA Vaccination. mBio.

[22] Mellis I.A., Wu M., Hong H., Tzang C.-C., Bowen A., Wang Q., Gherasim C., Pierce V.M., Shah J.G., Purpura L.J. (2025). Antibody Evasion and Receptor Binding of SARS-CoV-2 LP.8.1.1, NB.1.8.1, XFG, and Related Subvariants. Cell Rep..

