# Supplementary material for: Intra-Omicron Reinfection with JN.1.16 and NB.1.8.1 in a Preterm Infant: First NB.1.8.1 Detection in Tunisia—A Case Report
Source: Microorganisms. 2026 Apr 30;14(5):1009. doi: 10.3390/microorganisms14051009 (PMC13209800; doi:10.3390/microorganisms14051009)
Supplement: Supplementary file 1 [file microorganisms-14-01009-s001.zip › microorganisms-4054275-supplementary.pdf]

Supplementary Table S1. Key spike mutations and functional annotations in JN.1.16 (Episode 1) and NB.1.8.1 (Episode 3)

Mutation	Spike_region/domain	Variant(s)	Functional_category	Known / expected effect	Potential relevance in this case
A27S	N-terminal domain (NTD)	JN.1.16 & NB.1.8.1	NTD antigenic supersite	Escape from NTD-targeting nAbs; part of Omicron NTD remodeling	Shared in both episodes; may reduce recognition by pre-existing NTD antibodies and contribute to reinfection
ΔH69/ΔV70	NTD loop 69–70	JN.1.16 & NB.1.8.1	Fitness & antigenicity	Increases infectivity and spike incorporation; modulates NTD antigenicity	Could enhance upper-airway fitness and favor transmission/reinfection in a susceptible infant
G142D + ΔY144	NTD antigenic supersite	JN.1.16 & NB.1.8.1	Immune escape (NTD)	Strong reduction of binding by multiple NTD-directed nAbs	Supports escape from antibodies induced by prior infection or maternal antibodies
H245N	NTD	JN.1.16 & NB.1.8.1	Antigenicity/structural	Part of Omicron NTD remodeling; reshapes local antigenic surface	Adds to cumulative NTD antigenic drift between episodes
A264D	NTD (NB.1.8.1 only)	NB.1.8.1	NTD antigenic change	Alters charge in NTD; reported in NB sublineages;	Absent in JN.1.16, providing additional

				may fine-tune antigenicity	antigenic difference at reinfection
G339H	RBD (outer face)	NB.1.8.1	Immune escape (class 3 RBD Abs)	Reduces binding of some class 3 nAbs targeting lateral RBD surface	Adds RBD-level escape in NB.1.8.1 beyond JN.1.16
K356T	RBD core	NB.1.8.1	RBD antigenicity	Near class 1/2 epitopes; may modulate antibody binding and ACE2 affinity	Contributes to distinct RBD antigenic profile of NB.1.8.1
N440K	RBD (class 3 epitope)	JN.1.16	Immune escape (class 3 RBD Abs)	Reduces neutralization by several mAbs targeting lateral RBD surface	Present only in Episode 1; indicates different RBD escape pattern vs Episode 3
L452W	RBD, receptor-binding ridge	JN.1.16	Immune escape & fusogenicity	L452 substitutions (R/Q/W) associated with escape from T-cell and Ab responses; may increase fusogenicity	May have contributed to efficient infection and lung involvement during Episode 1

F456L	RBD, RBM	JN.1.16	Immune escape from XBB-era Abs	Key for escape from updated vaccine sera; modulates ACE2 binding	Further differentiates JN.1.16 escape profile from NB.1.8.1
N460K	RBD, RBM edge	JN.1.16	Immune escape & fusogenicity	Enhances syncytia formation and contributes to escape from several nAbs	Could relate to more pronounced lower-respiratory involvement in Episode 1
E484K	RBD, major antigenic site	JN.1.16	Strong immune escape	Classic escape mutation reducing neutralization by many sera	Key contributor to immune evasion of the first infecting virus
F486P	RBD, RBM	JN.1.16	Immune escape & ACE2 binding	Central to XBB/JN.1 family; confers broad escape while retaining ACE2 affinity	Supports extensive immune escape of JN.1.16; baseline from which NB.1.8.1 diverged
Q498R + N501Y + Y505H	RBD-ACE2 interface	JN.1.16	Enhanced ACE2 affinity & immune escape	Cooperative effect increasing ACE2 affinity and partially offsetting costs of other escape mutations	Explains efficient infection despite heavy RBD remodeling

D614G	S1 (near SD2)	JN.1.16 & NB.1.8.1	Baseline fitness mutation	Increases spike stability and infectivity; fixed in nearly all lineages	Background mutation; not specific to reinfection but part of high-fitness backbone
H655Y	Near S1/S2 site	JN.1.16 & NB.1.8.1	Entry/fusion	Improves spike processing and can shift entry toward endosomal route in Omicron	May facilitate efficient entry in infant respiratory epithelium
N679K + P681R	Furin cleavage loop	JN.1.16 & NB.1.8.1	Enhanced S1/S2 cleavage	Increase polybasic site efficiency, promoting S1/S2 cleavage and membrane fusion (context-dependent)	Could favor deeper lung infection; relevant in severe episodes
A688T	Proximal to cleavage site (NB.1.8.1)	NB.1.8.1	Fine-tuning of cleavage/fusion	Additional change in S1/S2 region; may modulate fusion and antigenicity	Unique to NB.1.8.1, adding divergence around the cleavage site
Q954H + N969K	S2, HR1–HR2 region	JN.1.16 & NB.1.8.1	Fusion machinery	Omicron-defining S2 changes; modulate fusion kinetics and	Part of common Omicron S2 scaffold; relevant to pathogenesis

				sensitivity to some S2-targeting Abs	
P1143L	S2 stem-helix / HR2 (ectodomain, membrane-proximal)	JN.1.16 & NB.1.8.1	Spike stability/fusion	Conserved stem-helix region targeted by broad S2 nAbs; local changes can affect spike stability, fusion and S2-directed antibody recognition	Background S2 change present in both episodes
K1149R	S2 stem-helix / HR2 (ectodomain, membrane-proximal)	NB.1.8.1	Spike stability/fusion	Same stem-helix context as above; potential effect on spike incorporation and S2-nAb recognition	Additional NB.1.8.1 change further differentiating the second infection

Δ (del), deletion; NTD, N-terminal domain; RBD, receptor-binding domain; RBM, receptor-binding motif; S1/S2, Spike subunits 1 and 2; HR1/HR2, heptad repeat 1 and 2; ACE2, angiotensin-converting enzyme 2; nAb(s), neutralizing antibody(ies); mAb(s), monoclonal antibody(ies); Ab(s), antibody(ies).

Supplementary Table S2. Tunisian SARS-CoV-2 Omicron genomes included in the phylogenetic analysis (GISAID metadata; n = 106, 1 November 2021–30 November 2025)

Virus name	Accession ID	Collection date	Location	Host	Sampling strategy	Gender	Patient age	Patient status	Passage	Specimen	Lineage	Clade
hCoV-19/Tunisia/CNH-64812/2021	EPI_ISL_15022542	30/12/2021	Africa / Tunisia / Tunis	Human	Baseline surveillance	Male	35	unknown	Original	Nasopharyngeal swab	BA.1	GRA
hCoV-19/Tunisia/F-7846/2022	EPI_ISL_16847407	11/01/2022	Africa / Tunisia / Tunis	Human	Random	Female	13	unknown	Original	Nasopharyngeal swab	BA.1.1.1	GRA
hCoV-19/env/Tunisia/UM-S1/2021	EPI_ISL_19615741	29/12/2021	Africa / Tunisia	Environment		unknown	unknown	unknown	Original		BA.1.1.12	GRA
hCoV-19/Tunisia/IPT-S-2857/2022	EPI_ISL_17731616	06/04/2022	Africa / Tunisia / Gafsa	Human	Random	Female	unknown	unknown	Original	Nasopharyngeal swab	BA.1.1.15	GRA
hCoV-19/Tunisia/S-2539/2022	EPI_ISL_16185975	25/01/2022	Africa / Tunisia / Kairouan	Human	Random	Male	unknown	unknown	Original	Nasopharyngeal swab	BA.1.13	GRA
hCoV-19/Tunisia/S-2514/2022	EPI_ISL_16185962	14/01/2022	Africa / Tunisia / Manouba	Human	Random	Female	25	unknown	Original	Nasopharyngeal swab	BA.1.15	GRA
hCoV-19/Tunisia/CNH-8148/2022	EPI_ISL_10863114	07/02/2022	Africa / Tunisia / Menzil Tmim	Human	Baseline surveillance	Male	53	unknown	Original	Nasopharyngeal swab	BA.1.17.2	GRA
hCoV-19/Tunisia/G-1447/2022	EPI_ISL_16185837	28/01/2022	Africa / Tunisia / Tunis	Human	Random	Female	71	unknown	Original	Nasopharyngeal swab	BA.1.18	GRA
hCoV-19/Tunisia/F-7255/2022	EPI_ISL_16185803	06/01/2022	Africa / Tunisia / Tunis	Human	Random	Male	34	unknown	Original	Nasopharyngeal swab	BA.1.21.1	GRA
hCoV-19/Tunisia/CNH-4163/2022	EPI_ISL_15022525	14/04/2022	Africa / Tunisia / Manouba	Human	Baseline surveillance	Female	60	unknown	Original	Nasopharyngeal swab	BA.2	GRA
hCoV-19/Tunisia/CNH-361/2023	EPI_ISL_18226702	27/02/2023	Africa / Tunisia / Ariana	Human	Baseline surveillance	Female	37	unknown	Original	Nasopharyngeal swab	BA.2.1	GRA

hCoV-19/Tunisia/CNH-3592/2023	EPI_ISL_17771479	16/03/2023	Africa / Tunisia / Tunis	Human	Baseline surveillance	Female	74	unknown	Original	Nasopharyngeal swab	BA.2.10.1	GR
hCoV-19/Tunisia/S-2770/2022	EPI_ISL_16186021	14/03/2022	Africa / Tunisia / Sousse	Human	Random	Male	1	unknown	Original	Nasopharyngeal swab	BA.2.3	GRA
hCoV-19/Tunisia/G-2720/2022	EPI_ISL_16185849	17/02/2022	Africa / Tunisia / Tunis	Human	Random	Male	59	unknown	Original	Nasopharyngeal swab	BA.2.21	GRA
hCoV-19/Tunisia/CNH-604/2022	EPI_ISL_15298425	04/07/2022	Africa / Tunisia / Kef	Human	Baseline surveillance	Female	40	unknown	Original	Nasopharyngeal swab	BA.2.36	GRA
hCoV-19/Tunisia/IPT-S-2884/2022	EPI_ISL_17731636	06/04/2022	Africa / Tunisia / Tunis	Human	Random	Male	unknown	unknown	Original	Nasopharyngeal swab	BA.2.32	GRA
hCoV-19/Tunisia/S-2637/2022	EPI_ISL_16186001	23/03/2022	Africa / Tunisia / Tunis	Human	Random	Female	83	unknown	Original	Nasopharyngeal swab	BA.2.5	GRA
hCoV-19/Tunisia/IPT-S-2859/2022	EPI_ISL_17731618	06/04/2022	Africa / Tunisia / Silina	Human	Random	Male	84	unknown	Original	Nasopharyngeal swab	BA.2.40.1	GRA
hCoV-19/Tunisia/IPT-S-2866/2022	EPI_ISL_17731625	06/04/2022	Africa / Tunisia / Sfax	Human	Random	Male	66	unknown	Original	Nasopharyngeal swab	BA.2.57	GRA
hCoV-19/Tunisia/IPT-S-2877/2022	EPI_ISL_17731634	06/04/2022	Africa / Tunisia / Tunis	Human	Random	Female	42	unknown	Original	Nasopharyngeal swab	BA.2.65	GRA
hCoV-19/Tunisia/CNH-S1023/2022	EPI_ISL_18877381	02/12/2022	Africa / Tunisia / Sfax	Human		Male	unknown	unknown	Original	Nasopharyngeal swab	BA.2.75	GRA
hCoV-19/Tunisia/G-3331/2022	EPI_ISL_16185894	16/03/2022	Africa / Tunisia / Tunis	Human	Random	Female	38	unknown	Original	Nasopharyngeal swab	BA.2.9	GRA
hCoV-19/Tunisia/CNH-S839/2022	EPI_ISL_15022571	24/06/2022	Africa / Tunisia / Sfax	Human	Baseline surveillance	Male	57	unknown	Original	Nasopharyngeal swab	BA.4	GRA
hCoV-19/Tunisia/V-6869/2022	EPI_ISL_16377417	01/08/2022	Africa / Tunisia / Tunis	Human	Random	Male	53	unknown	Original	Nasopharyngeal swab	BA.5	GRA
hCoV-19/Tunisia/V-6810/2022	EPI_ISL_16377414	26/07/2022	Africa / Tunisia / Tunis	Human	Random	Male	79	unknown	Original	Nasopharyngeal swab	BA.5.1.10	GRA

hCoV-19/Tunisia/CNH-14851/2022	EPI_ISL_15298475	30/05/2022	Africa / Tunisia / Tunis	Human	Baseline surveillance	Male	14	unknown	Original	Nasopharyngeal swab	BA.5.1	GRA
hCoV-19/Tunisia/CNH-S987/2022	EPI_ISL_16862238	15/09/2022	Africa / Tunisia / Sfax	Human	Baseline surveillance	Male	18	unknown	Original	Nasopharyngeal swab	BA.5.2	GRA
hCoV-19/Tunisia/CNH-473/2022	EPI_ISL_15022529	06/01/2022	Africa / Tunisia / Tunis	Human	Baseline surveillance	Female	37	unknown	Original	Nasopharyngeal swab	BA.1.1	GRA
hCoV-19/Tunisia/CNH-S942/2022	EPI_ISL_15022237	01/08/2022	Africa / Tunisia / Sfax	Human	Non-sentinel-surveillance (hospital)	Female	67	Hospitalized	Original	Nasopharyngeal swab	BA.5.2.20	GRA
hCoV-19/Tunisia/V-6929/2022	EPI_ISL_16186065	11/08/2022	Africa / Tunisia / Tunis	Human	Random	Female	77	unknown	Original	Nasopharyngeal swab	BA.5.2.44	GRA
hCoV-19/Tunisia/CNH-S1019/2022	EPI_ISL_16862260	17/11/2022	Africa / Tunisia / Sfax	Human	Baseline surveillance	Male	59	unknown	Original	Nasopharyngeal swab	BE.1.1.1	GRA
hCoV-19/Tunisia/CNH-562/2022	EPI_ISL_15298414	02/07/2022	Africa / Tunisia / Kef	Human	Baseline surveillance	Female	39	unknown	Original	Nasopharyngeal swab	BE.1.1	GRA
hCoV-19/Tunisia/CNH-570/2022	EPI_ISL_15298417	02/07/2022	Africa / Tunisia / Kef	Human	Baseline surveillance	Female	56	unknown	Original	Nasopharyngeal swab	BE.1	GRA
hCoV-19/Tunisia/CNH-2847/2023	EPI_ISL_17762985	02/03/2023	Africa / Tunisia / Sfax	Human	Baseline surveillance	Male	36	unknown	Original	Nasopharyngeal swab	BE.9	GRA
hCoV-19/Tunisia/CNH-S1021/2022	EPI_ISL_16862262	24/11/2022	Africa / Tunisia / Sfax	Human	Baseline surveillance	Female	74	unknown	Original	Nasopharyngeal swab	BN.1.3.7	GRA
hCoV-19/Tunisia/CNH-S1017/2022	EPI_ISL_16862258	15/11/2022	Africa / Tunisia / Sfax	Human	Baseline surveillance	Male	51	unknown	Original	Nasopharyngeal swab	BQ.1	GRA

hCoV-19/Tunisia/CNH-4160/2023	EPI_ISL_17764792	24/03/2023	Africa / Tunisia / Medenine	Human	Baseline surveillance	Male	55	unknown	Original	Nasopharyngeal swab	BN.1.4.2	GRA
hCoV-19/Tunisia/CNH-S1069/2023	EPI_ISL_18877383	23/02/2023	Africa / Tunisia / Sfax	Human		Female	51	unknown	Original	Nasopharyngeal swab	CH.1.1	GRA
hCoV-19/Tunisia/CNH-199/2023	EPI_ISL_18220463	04/02/2023	Africa / Tunisia / Ariana	Human	Baseline surveillance	Female	58	unknown	Original	Nasopharyngeal swab	CM.8.1	GRA
hCoV-19/Tunisia/CNH-S1104/2023	EPI_ISL_18877404	16/05/2023	Africa / Tunisia / Sfax	Human		Male	79	unknown	Original	Nasopharyngeal swab	EG.13	GRA
hCoV-19/Tunisia/CNH-483/2023	EPI_ISL_18227535	26/08/2023	Africa / Tunisia / Sousse	Human	Baseline surveillance	Female	27	unknown	Original	Nasopharyngeal swab	EG.1	GRA
hCoV-19/Tunisia/CNH-3184/2023	EPI_ISL_17764148	09/03/2023	Africa / Tunisia / Gabes	Human	Baseline surveillance	Female	20	unknown	Original	Nasopharyngeal swab	EG.4	GRA
hCoV-19/Tunisia/CNH-59881/2023	EPI_ISL_18161876	18/08/2023	Africa / Tunisia / Tunis	Human	Baseline surveillance	Female	36	unknown	Original	Nasopharyngeal swab	EG.2	GRA
hCoV-19/Tunisia/CNH-S1135/2023	EPI_ISL_18877420	22/09/2023	Africa / Tunisia / Sidi Bouzid	Human		Female	25	unknown	Original	Nasopharyngeal swab	EG.4.5	GRA
hCoV-19/Tunisia/CNH-10951/2023	EPI_ISL_18762075	10/11/2023	Africa / Tunisia / Jendouba	Human	Baseline surveillance	Male	unknown	unknown	Original	Nasopharyngeal swab	EG.5.1.1	GRA
hCoV-19/Tunisia/CNH-S1136/2023	EPI_ISL_18877421	22/09/2023	Africa / Tunisia / Sfax	Human		Male	79	unknown	Original	Nasopharyngeal swab	EG.5.1.3	GRA
hCoV-19/Tunisia/CNH-8438/2023	EPI_ISL_18762080	23/08/2023	Africa / Tunisia / Kef	Human	Baseline surveillance	Male	48	unknown	Original	Nasopharyngeal swab	FL.1	G

hCoV-19/Tunisia/CNH-8707/2023	EPI_ISL_18762495	31/08/2023	Africa / Tunisia / Tunis	Human	Baseline surveillance	Male	15	unknown	Original	Nasopharyngeal swab	FL.1.5.1	GRA
hCoV-19/Tunisia/CNH-S1097/2023	EPI_ISL_18877401	13/04/2023	Africa / Tunisia / Sfax	Human		Male	37	unknown	Original	Nasopharyngeal swab	FL.13	GRA
hCoV-19/Tunisia/CNH-528/2023	EPI_ISL_18220470	30/03/2023	Africa / Tunisia / Ariana	Human	Baseline surveillance	Male	70	unknown	Original	Nasopharyngeal swab	FL.10	GRA
hCoV-19/Tunisia/CNH-S1139/2023	EPI_ISL_18877424	05/10/2023	Africa / Tunisia / Sfax	Human		Female	30	unknown	Original	Nasopharyngeal swab	FL.24	GR
hCoV-19/Tunisia/CNH-2907/2023	EPI_ISL_17762991	02/03/2023	Africa / Tunisia / Nabeul	Human	Baseline surveillance	Male	40	unknown	Original	Nasopharyngeal swab	FL.2	GRA
hCoV-19/Tunisia/CNH-234687/2023	EPI_ISL_18226720	31/07/2023	Africa / Tunisia / Tunis	Human	Baseline surveillance	Male	64	unknown	Original	Nasopharyngeal swab	FL.25	GRA
hCoV-19/Tunisia/CNH-S1096/2023	EPI_ISL_18877400	12/04/2023	Africa / Tunisia / Sfax	Human		Female	59	unknown	Original	Nasopharyngeal swab	FL.4	GRA
hCoV-19/Tunisia/CNH-11209/2023	EPI_ISL_18762079	17/11/2023	Africa / Tunisia / Jendouba	Human	Baseline surveillance	Male	88	unknown	Original	Nasopharyngeal swab	FY.5	GRA
hCoV-19/Tunisia/CNH-11191/2023	EPI_ISL_18762498	17/11/2023	Africa / Tunisia / Kebili	Human	Baseline surveillance	Female	24	unknown	Original	Nasopharyngeal swab	GS.4	GRA
hCoV-19/Tunisia/CNH-12677/2023	EPI_ISL_18885649	27/12/2023	Africa / Tunisia / Tunis	Human	Baseline surveillance	Male	3 months	unknown	Original	Nasopharyngeal swab	GS.4.1	GRA
hCoV-19/Tunisia/CNH-11812/2023	EPI_ISL_18860288	01/12/2023	Africa / Tunisia / Sousse	Human	Baseline surveillance	Female	33	unknown	Original	Nasopharyngeal swab	HV.1	GRA

hCoV-19/Tunisia/CNH-8853/2023	EPI_ISL_18762067	06/09/2023	Africa / Tunisia / Nabeul	Human	Baseline surveillance	Male	5 months	unknown	Original	Nasopharyngeal swab	JG.2	GRA
hCoV-19/Tunisia/CNH-12723/2023	EPI_ISL_18885542	27/12/2023	Africa / Tunisia / Sidi Bouzid	Human	Baseline surveillance	Female	3	unknown	Original	Nasopharyngeal swab	JN.1	GRA
hCoV-19/Tunisia/CNH-12075/2023	EPI_ISL_18860289	08/12/2023	Africa / Tunisia / Kebili	Human	Baseline surveillance	Female	50	unknown	Original	Nasopharyngeal swab	JG.3	GRA
hCoV-19/Tunisia/CNH-881/2024	EPI_ISL_18885537	23/01/2024	Africa / Tunisia / Sidi Bouzid	Human	Baseline surveillance	Male	57	unknown	Original	Nasopharyngeal swab	JN.1.1.1	GRA
hCoV-19/Tunisia/CNH-645/2024	EPI_ISL_18885535	18/01/2024	Africa / Tunisia / Tunis	Human	Baseline surveillance	Female	2 months	unknown	Original	Nasopharyngeal swab	JN.1.1	GRA
hCoV-19/Tunisia/CNH-1342/2024	EPI_ISL_18941904	30/01/2024	Africa / Tunisia / Sfax	Human	Baseline surveillance	Female	34	unknown	Original	Nasopharyngeal swab	JN.1.1.10	GRA
hCoV-19/Tunisia/CNH-1596/2024	EPI_ISL_18941908	30/01/2024	Africa / Tunisia / Sfax	Human	Baseline surveillance	Male	51	unknown	Original	Nasopharyngeal swab	JN.1.10 (consensus call)	G
hCoV-19/Tunisia/10275/2024	EPI_ISL_19704729	05/11/2024	Africa / Tunisia / Ariana	Human	Baseline surveillance	Female	27	unknown	Original	Nasopharyngeal swab	JN.1.15 (consensus call)	GRA
hCoV-19/Tunisia/CNH-9251/2024	EPI_ISL_19696895	02/10/2024	Africa / Tunisia / Ariana	Human	Baseline surveillance	Female	78	unknown	Original	Nasopharyngeal swab	JN.1.11	GRA
hCoV-19/Tunisia/6272/2025	EPI_ISL_20142345	05/07/2025	Africa / Tunisia / Tunis	Human	Baseline surveillance	Male	3 months	unknown	Original	Nasopharyngeal swab	JN.1.16 (consensus call)	GRA
hCoV-19/Tunisia/CNH-8044/2024	EPI_ISL_19409927	19/08/2024	Africa / Tunisia / Tunis	Human	Baseline surveillance	Female	unknown	unknown	Original	Nasopharyngeal swab	JN.1.16.1 (consensus call)	GRA

hCoV-19/Tunisia/CNH-7992/2024	EPI_ISL_19409923	19/08/2024	Africa / Tunisia / Ariana	Human	Baseline surveillance	Male	65	unknown	Original	Nasopharyngeal swab	JN.1.18.5 (consensus call)	GRA
hCoV-19/Tunisia/CNH-3502/2024	EPI_ISL_19140066	21/03/2024	Africa / Tunisia / Kasserine	Human	Baseline surveillance	Female	57	unknown	Original	Nasopharyngeal swab	JN.1.17 (consensus call)	GRA
hCoV-19/Tunisia/CNH-1704/2024	EPI_ISL_18941910	03/02/2024	Africa / Tunisia / Tunis	Human	Baseline surveillance	Female	54	unknown	Original	Nasopharyngeal swab	JN.1.4	GRA
hCoV-19/Tunisia/CNH-4622/2024	EPI_ISL_19140070	18/04/2024	Africa / Tunisia / Ariana	Human	Baseline surveillance	unknown	78	unknown	Original	Nasopharyngeal swab	JN.1.32 (consensus call)	GRA
hCoV-19/Tunisia/5696/2025	EPI_ISL_20142341	13/06/2025	Africa / Tunisia / Zaghouan	Human	Baseline surveillance	Male	62	unknown	Original	Nasopharyngeal swab	JN.1.40 (consensus call)	GRA
hCoV-19/Tunisia/CNH-2370/2024	EPI_ISL_19140065	17/02/2024	Africa / Tunisia / Sfax	Human	Baseline surveillance	unknown	51	unknown	Original	Nasopharyngeal swab	JN.1.4.7 (consensus call)	GRA
hCoV-19/Tunisia/CNH-6966/2024	EPI_ISL_19409919	12/07/2024	Africa / Tunisia / Ariana	Human	Baseline surveillance	Male	60	unknown	Original	Nasopharyngeal swab	JN.1.48.1 (consensus call)	GRA
hCoV-19/Tunisia/CNH-1035/2024	EPI_ISL_18860285	26/01/2024	Africa / Tunisia / Sfax	Human	Baseline surveillance	Female	65	unknown	Original	Nasopharyngeal swab	JN.1.59	GRA
hCoV-19/Tunisia/CNH-1394/2024	EPI_ISL_18941905	26/01/2024	Africa / Tunisia / Nabeul	Human	Baseline surveillance	Female	70	unknown	Original	Nasopharyngeal swab	JN.1.67	GRA
hCoV-19/Tunisia/CNH-719/2024	EPI_ISL_18885536	19/01/2024	Africa / Tunisia / Monastir	Human	Baseline surveillance	Female	62	unknown	Original	Nasopharyngeal swab	JN.1.64	GRA
hCoV-19/Tunisia/CNH-11879/2024	EPI_ISL_19696466	19/12/2024	Africa / Tunisia / Tunis	Human	Baseline surveillance	Female	unknown	unknown	Original	Nasopharyngeal swab	JN.1.8 (consensus call)	GRA

hCoV-19/Tunisia/CNH-12782/2023	EPI_ISL_18885543	30/12/2023	Africa / Tunisia / Sousse	Human	Baseline surveillance	Male	unknown	unknown	Original	Nasopharyngeal swab	JN.1.9 (consensus call)	GRA
hCoV-19/Tunisia/CNH-7999/2024	EPI_ISL_19409924	19/08/2024	Africa / Tunisia / Sfax	Human	Baseline surveillance	Male	73	unknown	Original	Nasopharyngeal swab	JN.11 (consensus call)	G
hCoV-19/Tunisia/CNH-7399/2024	EPI_ISL_19409921	26/07/2024	Africa / Tunisia / Sousse	Human	Baseline surveillance	Male	unknown	unknown	Original	Nasopharyngeal swab	KP.1.1.3 (consensus call)	GRA
hCoV-19/Tunisia/CNH-4868/2024	EPI_ISL_19140074	28/04/2024	Africa / Tunisia / Sidi Bouzid	Human	Baseline surveillance	unknown	43	unknown	Original	Nasopharyngeal swab	LA.1 (consensus call)	GRA
hCoV-19/Tunisia/CNH-8041/2024	EPI_ISL_19409926	19/08/2024	Africa / Tunisia / Tunis	Human	Baseline surveillance	Female	64	unknown	Original	Nasopharyngeal swab	KP.3.1 (consensus call)	GRA
hCoV-19/Tunisia/8161/2024	EPI_ISL_19704725	23/08/2024	Africa / Tunisia / Ariana	Human	Baseline surveillance	Male	unknown	unknown	Original	Nasopharyngeal swab	KS.1.1 (consensus call)	GRA
hCoV-19/Tunisia/2532/2025	EPI_ISL_20142337	05/03/2025	Africa / Tunisia / Bizerte	Human	Baseline surveillance	Male	62	unknown	Original	Nasopharyngeal swab	LF.7.1 (consensus call)	GRA
hCoV-19/Tunisia/10886/2024	EPI_ISL_19704732	22/11/2024	Africa / Tunisia / Tunis	Human	Baseline surveillance	Female	73	unknown	Original	Nasopharyngeal swab	LF.7.1.2 (consensus call)	GRA
hCoV-19/Tunisia/9651/2024	EPI_ISL_19704727	16/10/2024	Africa / Tunisia / Jendouba	Human	Baseline surveillance	Male	84	unknown	Original	Nasopharyngeal swab	MC.42 (consensus call)	GRA
hCoV-19/Tunisia/6156/2025	EPI_ISL_20142343	30/06/2025	Africa / Tunisia / Gabes	Human	Baseline surveillance	Male	42	unknown	Original	Nasopharyngeal swab	NY.3.2 (consensus call)	GRA
hCoV-19/Tunisia/CNH-4853/2024	EPI_ISL_19140073	29/04/2024	Africa / Tunisia / Ariana	Human	Baseline surveillance	unknown	22	unknown	Original	Nasopharyngeal swab	MD.1.1 (consensus call)	GRA

hCoV-19/Tunisia/11378/2024	EPI_ISL_19704733	05/12/2024	Africa / Tunisia / Tunis	Human	Baseline surveillance	Female	unknown	unknown	Original	Nasopharyngeal swab	MD.1.1.2 (consensus call)	GRA
hCoV-19/Tunisia/5698/2025	EPI_ISL_20142330	13/06/2025	Africa / Tunisia / Ariana	Human	Baseline surveillance	Female	unknown	unknown	Original	Nasopharyngeal swab	PL.1 (consensus call)	GRA
hCoV-19/Tunisia/5896/2025	EPI_ISL_20142331	21/06/2025	Africa / Tunisia / Sfax	Human	Baseline surveillance	Female	56	unknown	Original	Nasopharyngeal swab	PY.1 (consensus call)	GRA
hCoV-19/Tunisia/CNH-405/2023	EPI_ISL_18226705	08/03/2023	Africa / Tunisia / Ariana	Human	Baseline surveillance	Female	37	unknown	Original	Nasopharyngeal swab	XBB	GRA
hCoV-19/Tunisia/CNH-S1066/2023	EPI_ISL_18877382	08/02/2023	Africa / Tunisia / Sfax	Human		Female	27	unknown	Original	Nasopharyngeal swab	XBB.1	GRA
hCoV-19/Tunisia/CNH-S1020/2022	EPI_ISL_16862261	19/11/2022	Africa / Tunisia / Sfax	Human	Baseline surveillance	Male	63	unknown	Original	Nasopharyngeal swab	XBB.1.9	GRA
hCoV-19/Tunisia/CNH-373/2023	EPI_ISL_18227526	12/08/2023	Africa / Tunisia / Sousse	Human	Baseline surveillance	Male	1 month	unknown	Original	Nasopharyngeal swab	XBB.1.16	GRA
hCoV-19/Tunisia/CNH-2269/2023	EPI_ISL_17762858	16/02/2023	Africa / Tunisia / Sfax	Human	Baseline surveillance	Male	63	unknown	Original	Nasopharyngeal swab	XBB.1.5	GRA
hCoV-19/Tunisia/CNH-S1138/2023	EPI_ISL_18877423	29/09/2023	Africa / Tunisia / Sfax	Human		Male	6 months	unknown	Original	Nasopharyngeal swab	XBB.2.3.11	GRA
hCoV-19/Tunisia/CNH-5984/2024	EPI_ISL_19409918	06/07/2024	Africa / Tunisia / Tunis	Human	Baseline surveillance	Male	1 month	unknown	Original	Nasopharyngeal swab	XDK (consensus call)	GRA
hCoV-19/Tunisia/6249/2025	EPI_ISL_20142344	03/07/2025	Africa / Tunisia / Tunis	Human	Baseline surveillance	Male	2 months	unknown	Original	Nasopharyngeal swab	XFG (consensus call)	GRA

hCoV-19/Tunisia/CNH-2144/2024	EPI_ISL_19140063	19/02/2024	Africa / Tunisia / Tunis	Human	Baseline surveillance	Female	40	unknown	Original	Nasopharyngeal swab	XDQ (consensus call)	GRA
hCoV-19/Tunisia/7817/2025	EPI_ISL_20207114	25/08/2025	Africa / Tunisia / Le Kef	Human	Baseline surveillance	Male	45	unknown	Original	Nasopharyngeal swab	XFG.3.1 (consensus call)	GRA
hCoV-19/Tunisia/7592/2025	EPI_ISL_20207111	20/08/2025	Africa / Tunisia / Tunis	Human	Baseline surveillance	Male	3 months	unknown	Original	Nasopharyngeal swab	XDV (consensus call)	GRA
hCoV-19/Tunisia/CNH-S796/2022	EPI_ISL_15022564	08/06/2022	Africa / Tunisia / Sfax	Human	Baseline surveillance	Male	59	unknown	Original	Nasopharyngeal swab	BA.5.2.1	GRA
